# Genetic Polymorphisms of Vascular Endothelial Growth Factor and Their Impact on Recurrent Spontaneous Miscarriage in Saudi Women

**DOI:** 10.3390/ijms26104757

**Published:** 2025-05-16

**Authors:** Wadha Khalid Al-Qahtani, Afrah Fahad Alkhuriji, Zeneb Ahmed Babay, Aaishah Mohammed Hussain Kaabi, Nawal M. Al-Malahi, Jamilah Obaid Alshammari

**Affiliations:** 1 Department of Zoology, College of Science, King Saud University, Riyadh 11451, Saudi Arabia; 444203101@student.ksu.edu.sa (W.K.A.-Q.); aalkhuriji@ksu.edu.sa (A.F.A.); jalshammary@ksu.edu.sa (J.O.A.); 2 Department of Obstetrics and Gynaecology, King Khalid University Hospital, King Saud University, Riyadh 11461, Saudi Arabia; zbabay@hotmail.com; 3 Department of Biology, College of Science, Qassim University, Buraidah 51452, Saudi Arabia; kaby@qu.edu.sa

**Keywords:** vascular endothelial growth factor (*VEGF*), single-nucleotide polymorphisms, recurrent spontaneous miscarriage

## Abstract

Recurrent spontaneous miscarriage (RSM) is defined as the loss of three or more clinically recognized pregnancies before 20 weeks of gestation. Angiogenesis, a crucial process in early pregnancy, is regulated by vascular endothelial growth factor (VEGF), a protein that plays a pivotal role in successful pregnancy. Disruptions in vascular development, such as those due to variations in *VEGF* gene expression, may contribute to infertility and pregnancy complications. Therefore, there is a need for more studies that show the effect of *VEGF* on RSM. This study investigated the impact of *VEGF* gene polymorphisms on RSM in Saudi women. Blood samples were collected from 200 Saudi women (100 cases with RSM and 100 controls). DNA was extracted from the buffy coat and analyzed for *VEGF* polymorphisms (rs10434, rs3025053, rs699947, rs2010963, rs833061, and rs25648) using TaqMan Real-Time PCR. Plasma VEGF levels were measured using the Human VEGF ELISA Kit. There was no significant association between rs10434, rs833061, and rs25648 and RSM. However, rs2010963, rs3025053, and rs699947 were significantly associated with an increased risk of miscarriage (*p* < 0.05). Furthermore, VEGF concentrations were significantly lower in the RSM case group (both pregnant and non-pregnant) compared to the control group (*p* < 0.05). *VEGF* polymorphisms, along with reduced *VEGF* serum levels, are associated with an increased risk of RSM in Saudi women. Further studies are needed to explore the underlying mechanisms and potential therapeutic targets.

## 1. Introduction

Recurrent spontaneous miscarriage (RSM) is defined as three or more consecutive pregnancy losses before 20 weeks of gestation [1,2,3]. Genetic abnormalities are a leading cause of early pregnancy loss, with approximately 50% of first-trimester miscarriages exhibiting chromosomal abnormalities [4,5].

Various factors contribute to RSM, including immunological disorders, antiphospholipid syndrome, thrombophilia, smoking, alcohol consumption, age, environmental pollutants, radiation exposure, uterine abnormalities, infections, and endocrine imbalances [6,7]. RSM is associated with adverse pregnancy outcomes, including placental abruption, fetal growth restriction, stillbirth, and preterm birth [8].

Vascular endothelial growth factor (VEGF) is a widely recognized angiogenic factor that plays a crucial role in various physiological and pathological processes [9]. The vascular endothelial growth factor (*VEGF*) gene is located within a specific region of chromosome 6p21.3 and has a complex structural composition consisting of eight exons interspersed with seven introns [10].

*VEGF* is a key regulator of angiogenesis and is critical for embryo implantation, placental development, and endothelial cell function. Alterations in *VEGF* expression have been linked to recurrent implantation failure and RSM [11]. In 2021, a study was conducted to examine the relationships between recurrent implantation failure (RIF) and vascular endothelial growth factor (*VEGF*) polymorphisms. It was determined that women with *VEGF* 1154 AA/GA genotypes were at higher risk of recurrent implantation failure (RIF). The *VEGF*-1154A allele may serve as one of the predisposing factors of RIF [12]. In another study, patients with the ID genetic model of -2549I/D polymorphisms in the promoter region of the *VEGFA* gene, along with the presence of the D allele, are at a greater risk for idiopathic recurrent spontaneous miscarriage (IRSM) [13]. In one study conducted on Iranian women, researchers found that the *VEGF* coding gene polymorphism rs10434 significantly differed between the patient and control groups, and this variation may have an impact on the prevalence of pre-eclampsia (PE), which is considered a pregnancy complication [14].

Many studies have shown that certain vascular endothelial growth factor polymorphisms are associated with gastrointestinal tract cancer risk. Researchers have discovered a significant association between gallbladder cancer risk and *VEGF*-2578C/A polymorphism. On the other hand, *VEGF*-460T/C polymorphism was associated with higher risks of colorectal, gastric, and hepatocellular cancers [15]. Also, in another study, the results showed that according to the study’s findings, blood *VEGF* levels and the rs2010963 *VEGF* gene polymorphism may be risk factors for breast cancer. In comparison to patients with the CC genotype, individuals with the GG genotype had the highest serum *VEGF* levels, bigger tumors, more advanced tumor stages, and a shorter survival time [16]. In a study involving Pakistani patients, the results indicated a strong association between the rs699947 polymorphism and the risk of developing proliferative diabetic retinopathy (PDR) in type 2 diabetes patients [17].

An in vitro study investigated endothelial reactivity to *VEGF*, which showed that VEGF strongly induces in vitro angiogenesis in pregnant human uterine artery endothelial cells (P-hUAECs) but not in non-pregnant human uterine artery endothelial cells (NP-hUAECs) [18]. In humans, the *VEGF* family consists of various members, including *VEGF-A*, *VEGF-B*, *VEGF-C*, *VEGF-D*, and placental growth factor (*PlGF*) [19,20].

Tyrosine kinase cell receptors (VEGFRs) are bound by VEGF and include VEGFR-1 (also known as Fms-like tyrosine kinase 1 (Flt-1)), VEGFR-2 (also known as CD309, fetal liver kinase 1 (Flk-1), or kinase insert domain receptor (KDR)), and VEGFR-3 (also known as Fms-like tyrosine kinase 4 (Flt-4)). Endothelial cells contain the co-receptors neuropilin-1 (NP-1) and neuropilin-2 (NP-2), which regulate tyrosine kinase receptor function. It is important to note that the expression of both vascular endothelial growth factor (*VEGF*) and its receptors (VEGFRs) is not limited to endothelial cells; they are also expressed in various non-endothelial cell types [19]. Another receptor, the soluble form of VEGFR-1 (sFlt-1), is strongly associated with recurrent miscarriage [21], adverse pregnancy outcomes [22], and unexplained infertility [23,24]. This is linked to its ability to trap *PlGF*, *VEGF*, and *VEGF-B*, preventing them from attaching to membrane receptors [9].

It has been established that the most significant and powerful factor in angiogenesis is *VEGF-A*, also known as *VEGF. VEGF-B* has been documented to possess significant antioxidant properties through its ability to enhance the production of critical antioxidant enzymes. Moreover, *VEGF-C* and *VEGF-D* have essential roles in lymphangiogenesis. In addition, *VEGF-D* binds to VEGFR-2 and VEGFR-3 and can control angiogenesis, lymphangiogenesis, fibrogenesis, and apoptosis; *VEGF-C* binds to VEGFR-2 and VEGFR-3 to control angiogenesis, inflammation, lymphangiogenesis, apoptosis, and fibrogenesis [25,26,27].

VEGF exerts its effects through its receptors VEGFR-1, VEGFR-2, and VEGFR-3, and its activity is modulated by several genetic polymorphisms [19]. Numerous studies have examined the genetic association of polymorphisms of *VEGF*, especially rs10434, rs3025053, rs699947, rs2010963, rs833061, and rs25648, which have been reported to have an effect on miscarriage in women of ethnically diverse groups, but there are no studies that specifically investigate these particular SNPs and their impact on miscarriage in Saudi women. Therefore, the purpose of this study was to determine whether the susceptibility of Saudi women to RSM might be influenced by these polymorphisms mentioned above.

## 2. Results

### 2.1. Demographic Data of Subjects Included in This Study

Table 1a displays the demographic data of the pregnant women (patients vs. controls).

The body mass index (BMI) and number of children were similar in both groups (*p* > 0.05), while there was a significant difference in age and the number of pregnancies (*p* < 0.05).

The demographic information of the non-pregnant women (patients vs. controls) is shown in Table 1b.

Age and BMI were matched in both groups (*p* > 0.05); however, a significant disparity was seen in the number of pregnancies and the number of children (*p* < 0.05).

### 2.2. Genotypic and Allelic Frequencies

#### 2.2.1. rs699947, (-2578C/A)

The (-2578C/A) polymorphism in the promoter region of the vascular endothelial growth factor (*VEGF*) gene and wild-type homozygous (CC) forms were identified.

Table 2a presents the genotype and allele frequency of (-2578C/A) polymorphism in pregnant patients and pregnant controls. We observed no statistically significant association between the wild-type CC (*p* = 0.15) and AA (*p* = 0.48) genotype and RSM in pregnant Saudi women. However, the -2578 AC genotype was linked to a lower risk of RSM development and had an odds ratio (OR) of 0.43 in pregnant women, and the frequency between case and control is significant (*p* = 0.04).

There were no notable variations between the non-pregnant patients and non-pregnant control groups (Table 2b).

#### 2.2.2. rs2010963, (-634G/C)

Table 3a (pregnant, patients vs. controls) and Table 3b (non-pregnant, patients vs. controls) show the genotype and allele frequencies for the -634G/C (rs2010963) polymorphism in the 5′-untranslated region (5′-UTR) of the *VEGF* gene, and the wild type is homozygous (CC).

The results show that the rs2010963 (-634G/C) genotype wild-type homozygous CC (OR = 4.95, *p* = 0.02) was associated with the risk of recurrent spontaneous miscarriage, while the genotype GG (OR = 0.32, *p* = 0.01) showed less association with RSM. Additionally, there was a statistically significant difference (*p* = 0.001) in the frequencies of the G and C alleles for rs2010963 (-634G/C) in pregnant patients compared to pregnancy controls. However, there was no statistically significant difference in the genotype CG (*p* = 0.35) (Table 3a). There were no significant differences between the non-pregnant patient group and the relevant controls (Table 3b).

#### 2.2.3. rs3025053, (1725G/A)

The genotype and allele frequencies for the 1725G/A (rs3025053) polymorphism in the 3′-untranslated region (3′-UTR) of the *VEGF* gene are displayed in Table 4a for the pregnant group (patients vs. controls) and in Table 4b for the non-pregnant group (patients vs. controls). Most of the pregnant and non-pregnant women were homozygous for the wild-type genotype (GG).

The results show that the frequency of the genotypes wild-type GG and GA (*p* = 0.01) for rs3025053 (1725G>A) was statistically significantly different between the pregnant patient group and the pregnant control group. Furthermore, the adjusted OR for RSM for the GG genotype was 3.58, and that for the GA genotype was 0.26. Importantly, the allele frequencies (G and A) demonstrated a significant correlation with RSM (*p* = 0.02). For the genotype AA, there was no difference between the two groups of pregnant women (Table 4a).

Many patients and controls were homozygous for the wild-type genotype (GG); however, there were no significant differences between the non-pregnant patient and non-pregnant control groups (Table 4b).

#### 2.2.4. rs25648, rs833061, and rs10434

In contrast to the findings reported above, rs25648 (-7C>T) is located at the 5′-untranslated region (5′-UTR). As shown in Table 5a (pregnant) and Table 5b (non-pregnant), patients and controls showed higher frequencies of the homozygous wild-type genotype (CC). However, there were no statistically significant differences in either the pregnant (patients vs. controls) or non-pregnant (patients vs. controls) groups.

In addition, Table 6a (pregnant) and Table 6b (non-pregnant) present the polymorphism -460T>C (rs833061) located in the promoter region; the homozygous wild type was TT, and there were no significant differences between the pregnant and non-pregnant groups (patients vs. controls).

Also, the polymorphism rs10434 (1612G>A) was identified in the 3′-untranslated region (3′-UTR) of the *VEGF* gene. The homozygous wild-type genotype (GG) was more common in patients and controls in Table 7a (pregnant) and Table 7b (non-pregnant). Nevertheless, neither the pregnant (patients vs. controls) nor the non-pregnant (patients vs. controls) groups showed any statistically significant changes.

### 2.3. VEGF Plasma Levels

#### 2.3.1. Comparison of *VEGF* Plasma Levels in Pregnant Patients (Patients vs. Controls)

**Figure 1:**
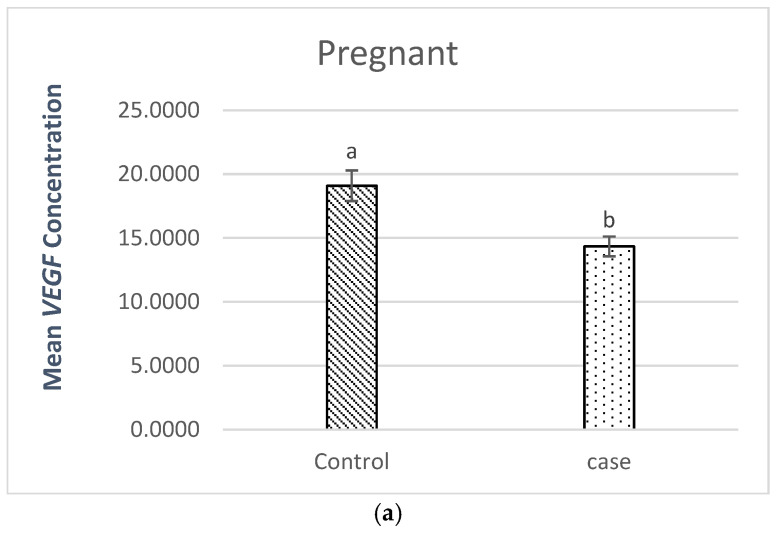

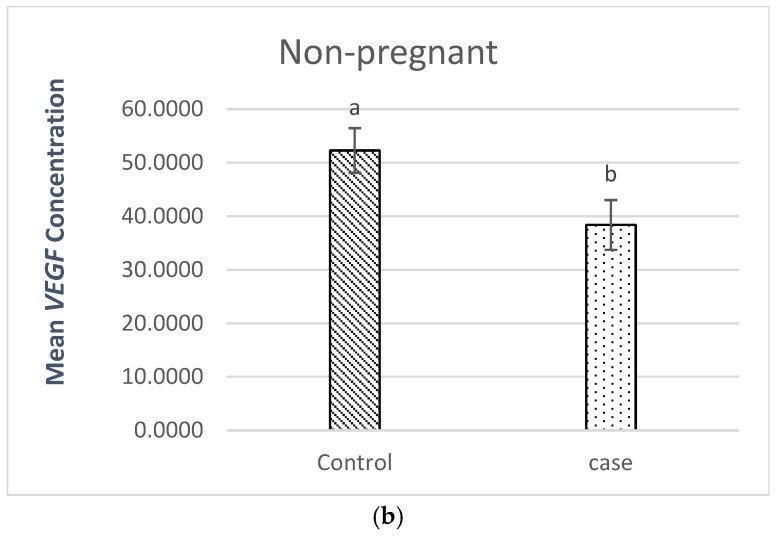
(**a**). Comparison of *VEGF* plasma levels in pregnant patients (patients vs. controls), where a, b stand for different superscripts within columns that are significantly different, *p* < 0.05. (**b**). Comparison of VEGF plasma levels in non-pregnant patients (patients vs. controls), where a, b stand for different superscripts within columns that are significantly different, *p* < 0.05.

Figure 1a presents the results of the comparison between pregnant women (patients vs. controls). The results showed a statistically significant difference between pregnant women (patients vs. controls), with a *p*-value = 0.003.

The level of *VEGF* plasma in the pregnant patients (14.34 pg/mL) was lower than the level (19.09 pg/mL) found in the pregnant controls.

#### 2.3.2. Comparison of *VEGF* Plasma Levels in Non-Pregnant Patients (Patients vs. Controls)

The comparison of non-pregnant women (patients vs. controls) is shown in Figure 1b. The results demonstrated a statistically significant difference in the *p*-value (*p* = 0.034) in non-pregnant women (patients vs. controls).

The non-pregnant patients’ *VEGF* plasma level (38.39 pg/mL) was lower than the non-pregnant controls’ level (52.28 pg/mL).

The observed lower *VEGF* levels in cases with RSM (pregnant and non-pregnant) suggest impaired angiogenesis as a contributing factor to recurrent spontaneous miscarriage.

## 3. Discussion

Vascular endothelial growth factor (*VEGF*) is recognized as an important growth factor due to its crucial pro-angiogenic properties. It exerts both a mitogenic influence and has an anti-apoptotic impact on endothelial cells. Moreover, *VEGF* is a multifunctional factor that controls vascular permeability and the proliferation, differentiation, and survival of endothelial cells [28].

Numerous single-nucleotide polymorphisms (SNPs) in the vascular endothelial growth factor (*VEGF*) gene have been documented, including rs699947 and rs833061, located in the promoter region of the *VEGF* gene [29]; rs3025053 and rs10434, situated in the 3′-untranslated region (3′-UTR) [30]; and rs2010963 and rs25648, found in the 5′-untranslated region (5′-UTR) [31,32], which are correlated with an elevated risk of miscarriage.

Our findings align with previous studies that reported increased prevalences of the rs2010963 C allele and rs699947 AC genotype in women with recurrent pregnancy loss [33,34]. Similar studies in Korean and Egyptian populations have also reported associations between *VEGF* polymorphisms and pregnancy complications [30,35]. However, some studies have reported conflicting results. For example, Elmi et al. (2023) found no significant association between rs2010963 and implantation failure [36].

In contrast, several studies involving Korean women have shown that 1612G>A polymorphisms are related to recurrent pregnancy loss (RPL) [30]. Additionally, the *VEGF* rs3025020/rs833061 genotype allele was found to be linked to recurrent pregnancy loss (RPL) and the levels of hematocrit (HCT) in the blood of RPL patients [37]. Moreover, the rs25648 T allele and the rs833061 C allele have been linked to recurrent implantation failure (RIF) [38].

Furthermore, Yalcintepe et al. found that when comparing fetal genotypes to their mothers and healthy controls, the *VEGF A* rs833061, rs2010963, and rs3025039 fetal genotypes are risk factors for spontaneous abortion [39]. In addition, *VEGF* expression and its relationship with recurrent spontaneous miscarriage (RSM) are affected by the *VEGFA* SNPs + 398G/A, 583T/C, and -460T/C, and there is also a weak correlation with 634G/C [40]. These discrepancies may be attributed to genetic differences across populations or variations in study design.

Deficiencies in the formation of blood vessels and blood vessel growth contribute to an increased risk of pregnancy loss [2]. Reduced *VEGF* levels have been implicated in pregnancy loss, as *VEGF* plays a critical role in vascular remodeling and endometrial receptivity [41]. Our results confirm a significant decrease in *VEGF* concentrations in RSM cases, supporting the hypothesis that *VEGF* deficiency contributes to pregnancy failure.

In a study to detect the level of *VEGF* in different stages of taken throughout pregnancy and postpartum, where blood samples were collected at 12, 20, 24, 28, 32, 36, and 40 gestational weeks, and in the period from 8 to 10 weeks after birth, the results showed that compared to the percentage seen 9–10 weeks postpartum (64%), the percentage of women with levels over the detection limit (9 pg/mL) in gestational weeks 9–10 (10%) and 39–40 (15%) was significantly lower. All samples obtained at 9–10 weeks postpartum had a median *VEGF-A* level of 76.4 pg/mL [42].

Furthermore, previous studies have observed elevated levels of *VEGF* in the serum of non-pregnant women compared to normal pregnant women during the third trimester of pregnancy [43]. In addition, another study noted that the level of *VEGF* in the plasma of the non-pregnant rats was significantly higher compared to the term pregnant ones [44].

Modifying the concentrations of growth factors has the potential to influence the intricate mechanisms involved in programmed cell death, cell division, and the formation of new blood vessels through angiogenesis. This could have profound implications on cellular functions and tissue development [45]. However, patients with various cardiovascular diseases (CVDs) have been discovered to have high levels of vascular endothelial growth factor-A (*VEGF-A*), which is commonly associated with adverse disease prognosis and severity [46]. An optimal level of *VEGF-A* is advantageous in preserving the structure of the glomerulus, whereas an excessive amount may result in aberrant angiogenesis [47].

## 4. Materials and Methods

### 4.1. The Study Design and Participants

This case–control study was conducted at King Khalid University Hospital, Riyadh, Saudi Arabia, and included 200 Saudi women aged 18–45 years. The case group consisted of 100 women (50 pregnant and 50 non-pregnant) with a history of unexplained recurrent spontaneous miscarriage (RSM). The control group included 100 healthy women (50 pregnant and 50 non-pregnant) with no history of miscarriage. All participants signed an informed consent form to participate in this study. The Medical Ethics Committee at King Khalid University Hospital and the Ethics Committee at King Saud University approved this study (protocol No. E-24-8865 and date of approval 15 July 2024). Women who had miscarriages with known causes, such as infections, anatomical abnormalities, hormonal imbalances, and chromosomal disorders, were excluded.

### 4.2. DNA Extraction and Genotyping

Five milliliters of blood was collected in EDTA tubes for DNA extraction using the GeneJET™ Whole Blood Genomic DNA Purification Mini Kit. Genotyping was performed using TaqMan SNP Genotyping Assays on the Real-Time PCR System (Applied Biosystems, Waltham, MA, USA). The genotyping assay targeted *VEGF* polymorphisms (rs10434, rs3025053, rs699947, rs2010963, rs833061, and rs25648).

### 4.3. Plasma VEGF Measurement

Two milliliters of blood were collected in sodium citrate tubes for plasma isolation. VEGF-A levels were measured using the Human *VEGF* ELISA Kit (Thermo Fisher Scientific, Catalog No. KHG0111, Waltham, MA, USA), which has an assay sensitivity of <5 pg/mL.

### 4.4. Statistical Analysis

Statistical analyses were performed using SPSS 22. The Chi-square test was used to compare genotype and allele frequencies. Independent t-tests were used to compare VEGF levels between groups. Odds ratios (ORs) and 95% confidence intervals (CIs) were calculated. A *p*-value < 0.05 was considered statistically significant.

## 5. Conclusions

The vascular endothelial growth factor (*VEGF*) polymorphisms rs699947, rs2010963, and rs3025053, along with reduced *VEGF* serum levels, are associated with an increased risk of RSM in Saudi women. In this study, no notable associations were found between the *VEGF* polymorphisms rs25648, rs10434, and rs833061 and recurrent spontaneous miscarriage.

Further investigations are imperative and should be conducted in order to thoroughly examine and elucidate the intricate underlying mechanisms that cause RSM, and identify and assess the potential therapeutic targets that may offer viable avenues for intervention and treatment.

## Figures and Tables

**Table 1:** (a). The demographic data of the pregnant women (patients vs. controls). (b). The demographic data of the non-pregnant women (patients vs. controls).

(a)			
**Parameter for Pregnant**	**Control**	**Case**	***p*-Value**
	**Mean ± S.E**		
Age (years)	33 ± 0.57	36 ± 0.73	0.002
Weight (kg)	75 ± 2.46	74 ± 1.83	0.668
Length (m)	2 ± 0.01	2 ± 0.01	0.755
Body mass index (kg/m^2^)	30 ±0.98	29 ± 0.71	0.727
No. of children	3 ± 0.14	3 ± 0.24	0.715
No. of abortions	0 ± 0.00	4 ± 0.29	0.000
(b)			
**Parameter for Non-Pregnant**	**Control**	**Case**	***p*-Value**
	**Mean ± S.E**		
Age (years)	35 ± 0.72	36 ± 0.78	0.499
Weight (kg)	69 ± 2.09	70 ± 1.78	0.765
Length (m)	2 ± 0.01	2 ± 0.01	0.117
Body mass index (kg/m^2^)	28 ± 0.79	28 ± 0.60	0.709
No. of children	3 ± 0.19	2 ± 0.25	0.000
No. of abortions	0 ± 0.00	5 ± 0.60	0.000

**Table 2:** (a). The genotype and allele frequencies of *VEGF* rs699947 (-2578C/A)—(pregnant). (b). The genotype and allele frequencies of *VEGF* rs699947 (-2578C/A)—(non-pregnant).

(a)						
**Genotype**	**Case** **Pregnant** ***N* = 50**	**Control** ***N* = 50**	**Case vs. Control**			
			**OR**	**CI**	**X^2^**	***p*-Value**
CC	22 (44%)	15 (30%)	1.83	0.80 to 4.18	2.08	*p* = 0.15
AC	15 (30%)	25 (50%)	0.43	0.19 to 0.98	4.13	*p* = 0.04
AA	13 (26%)	10 (20%)	1.41	0.55 to 3.59	0.50	*p* = 0.48
Total	50	50				
**Allele**	**Case** **(Freq)**	**Control (Freq)**	**Case vs. Control**			
			**OR**	**CI**	**X^2^**	***p*-Value**
C	59 (59%)	55 (55%)	1.18	0.67 to 2.06	0.32	*p* = 0.57
A	41 (41%)	45 (45%)	0.85	0.49 to 1.49		
(b)						
**Genotype**	**Case** ***N* = 50**	**Control** ***N* = 50**	**Case vs. Control**			
			**OR**	**CI**	**X^2^**	***p*-Value**
CC	15 (30%)	16 (32%)	0.91	0.39 to 2.13	0.05	*p* = 0.83
AC	30 (60%)	22 (44%)	1.91	0.86 to 4.23	2.54	*p* = 0.11
AA	5 (10%)	12 (24%)	0.35	0.11 to 1.09	3.44	*p* = 0.07
Total	50	50				
**Allele**	**Case** **(Freq)**	**Control (Freq)**	**Case vs. Control**			
			**OR**	**CI**	**X^2^**	***p*-Value**
C	60 (60%)	54 (54%)	1.28	0.73 to 2.24	0.73	*p* = 0.39
A	40 (40%)	46 (46%)	0.78	0.45 to 1.37		

**Table 3:** (a). The genotype and allele frequencies of *VEGF* rs2010963, (-634G/C)—(pregnant). (b). The genotype and allele frequencies of *VEGF* rs2010963, (-634G/C)—(non-pregnant).

(a)						
**Genotype**	**Case** **Pregnant** ***N* = 50**	**Control** ***N* = 50**	**Case vs. Control**			
			**OR**	**CI**	**X^2^**	***p*-Value**
CC	12 (24%)	3 (6%)	4.95	1.30 to 18.81	6.29	*p* = 0.02
CG	14 (28%)	10 (20%)	1.56	0.61 to 3.94	0.89	*p* = 0.35
GG	24 (48%)	37 (74%)	0.32	0.14 to 0.75	7.03	*p* = 0.01
Total	50	50				
**Allele**	**Case** **(Freq)**	**Control (Freq)**	**Case vs. Control**			
			**OR**	**CI**	**X^2^**	***p*-Value**
C	38 (38%)	16 (16%)	3.22	1.65 to 6.29	12.22	*p* = 0.001
G	62 (62%)	84 (84%)	0.31	0.16 to 0.61		
(b)						
**Genotype**	**Case** ***N* = 50**	**Control** ***N* = 50**	**Case vs. Control**			
			**OR**	**CI**	**X^2^**	***p*-Value**
CC	7 (14%)	9 (18%)	0.74	0.25 to 2.18	0.29	*p* = 0.59
CG	28 (56%)	21 (42%)	1.76	0.8 to 3.88	1.94	*p* = 0.16
GG	15 (30%)	20 (40%)	0.64	0.28 to 1.47	1.09	*p* = 0.3
Total	50	50				
**Allele**	**Case** **(Freq)**	**Control (Freq)**	**Case vs. Control**			
			**OR**	**CI**	**X^2^**	***p*-Value**
C	42 (42%)	39 (39%)	1.13	0.64 to 1.99	0.19	*p* = 0.67
G	58 (58%)	61 (61%)	0.88	0.50 to 1.55		

**Table 4:** (a). The genotype and allele frequencies of *VEGF* rs3025053, (1725G>A)—(pregnant). (b). The genotype and allele frequencies of *VEGF* rs3025053, (1725G>A)—(non-pregnant).

(a)						
**Genotype**	**Case** **pregnant** ***N* = 50**	**Control** ***N* = 50**	**Case vs. Control**			
			**OR**	**CI**	**X^2^**	***p*-Value**
GG	41 (82%)	28 (56%)	3.58	1.44 to 8.91	7.82	*p* = 0.01
GA	8 (16%)	21 (42%)	0.26	0.10 to 0.67	8.13	*p* = 0.01
AA	1 (2%)	1 (2%)	1.00	0.06 to 16.44	0	*p* = 1.00
Total	50	50				
**Allele**	**Case** **(Freq)**	**Control (Freq)**	**Case vs. Control**			
			**OR**	**CI**	**X^2^**	***p*-Value**
G	90 (90%)	77 (77%)	2.69	1.21 to 6	6.10	*p* = 0.02
A	10 (10%)	23 (23%)	0.37	0.17 to 0.83		
(b)						
**Genotype**	**Case** ***N* = 50**	**Control** ***N* = 50**	**Case vs. Control**			
			**OR**	**CI**	**X^2^**	***p*-Value**
GG	44 (88%)	45 (90%)	0.81	0.23 to 2.87	0.10	*p* = 0.75
GA	5 (10%)	5 (10%)	1.00	0.27 to 3.69	0	*p* = 1.00
AA	1 (2%)	0	3.06	0.12 to 76.95	1	*p* = 0.5
Total	50	50				
**Allele**	**Case** **(Freq)**	**Control (Freq)**	**Case vs. Control**			
			**OR**	**CI**	**X^2^**	***p*-Value**
G	93 (93%)	95 (95%)	0.7	0.21 to 2.28	0.35	*p* = 0.55
A	7 (7%)	5 (5%)	1.43	0.44 to 4.67		

**Table 5:** (a). The genotype and allele frequencies of *VEGF* rs25648 (-7C>T)—(pregnant). (b). The genotype and allele frequencies of *VEGF* rs25648 (-7C>T)—(non-pregnant).

(a)						
**Genotype**	**Case** **Pregnant** ***N* = 50**	**Control** ***N* = 50**	**Case vs. Control**			
			**OR**	**CI**	**X^2^**	***p*-Value**
CC	34 (68%)	29 (58%)	1.54	0.68 to 3.49	1.06	*p* = 0.30
CT	14 (28%)	20 (40%)	0.58	0.25 to 1.35	1.59	*p* = 0.21
TT	2 (4%)	1 (2%)	2.04	0.18 to 23.27	0.34	*p* = 0.57
Total	50	50				
**Allele**	**Case** **(Freq)**	**Control (Freq)**	**Case vs. Control**			
			**OR**	**CI**	**X^2^**	***p*-Value**
C	82 (82%)	78 (78%)	1.28	0.64 to 2.58	0.5	*p* = 0.48
T	18 (18%)	22 (22%)	0.78	0.39 to 1.56		
(b)						
**Genotype**	**Case** ***N* = 50**	**Control** ***N* = 50**	**Case vs. Control**			
			**OR**	**CI**	**X^2^**	***p*-Value**
CC	38 (76%)	36 (72%)	1.23	0.50 to 3.02	0.21	*p* = 0.65
CT	12 (24%)	12 (24%)	1	0.4 to 2.50	0	*p* = 1
TT	0	2 (4%)	0.19	0.01 to 4.10	2.02	*p* = 0.29
Total	50	50				
**Allele**	**Case** **(Freq)**	**Control (Freq)**	**Case vs. Control**			
			**OR**	**CI**	**X^2^**	***p*-Value**
C	88 (88%)	84 (84%)	1.4	0.62 to 3.13	0.66	*p* = 0.42
T	12 (12%)	16 (16%)	0.72	0.32 to 1.60		

**Table 6:** (a). The genotype and allele frequencies of *VEGF* rs833061, (-460T>C)—(pregnant). (b). The genotype and allele frequencies of *VEGF* rs833061, (-460T>C)—(non-pregnant).

(a)						
**Genotype**	**Case** **Pregnant** ***N* = 50**	**Control** ***N* = 50**	**Case vs. Control**			
			**OR**	**CI**	**X^2^**	***p*-Value**
CC	13 (26%)	12 (24%)	1.11	0.45 to 2.75	0.05	*p* = 0.82
CT	22 (44%)	21 (42%)	1.09	0.49 to 2.4	0.04	*p* = 0.84
TT	15 (30%)	17 (34%)	0.83	0.36 to 1.93	0.18	*p* = 0.67
Total	50	50				
**Allele**	**Case** **(Freq)**	**Control (Freq)**	**Case vs. Control**			
			**OR**	**CI**	**X^2^**	***p*-Value**
C	48 (48%)	45 (45%)	1.13	0.65 to 1.97	0.18	*p* = 0.67
T	52 (52%)	55 (55%)	0.89	0.51 to 1.55		
(b)						
**Genotype**	**Case** ***N* = 50**	**Control** ***N* = 50**	**Case vs. Control**			
			**OR**	**CI**	**X^2^**	***p*-Value**
CC	7 (14%)	12 (24%)	0.52	0.18 to 1.44	1.61	*p* = 0.21
CT	28 (56%)	22 (44%)	1.62	0.74 to 3.57	1.43	*p* = 0.23
TT	15 (30%)	16 (32%)	0.91	0.39 to 2.13	0.05	*p* = 0.83
Total	50	50				
**Allele**	**Case** **(Freq)**	**Control (Freq)**	**Case vs. Control**			
			**OR**	**CI**	**X^2^**	***p*-Value**
C	42 (42%)	46 (46%)	0.85	0.49 to 1.49	0.32	*p* = 0.57
T	58 (58%)	54 (54%)	1.18	0.67 to 2.06		

**Table 7:** (a). The genotype and allele frequencies of VEGF rs10434, (1612G>A)—(pregnant). (b). The genotype and allele frequencies of *VEGF* rs10434, (1612G>A)—(non-pregnant).

(a)						
**Genotype**	**Case** **Pregnant** ***N* = 50**	**Control** ***N* = 50**	**Case vs. Control**			
			**OR**	**CI**	**X^2^**	***p*-Value**
GG	22 (44%)	27 (54%)	0.67	0.30 to 1.47	0.99	*p* = 0.32
GA	22 (44%)	19 (38%)	1.28	0.58 to 2.85	0.37	*p* = 0.54
AA	6 (12%)	4 (8%)	1.57	0.41 to 5.94	0.44	*p* = 0.51
Total	50	50				
**Allele**	**Case** **(Freq)**	**Control (Freq)**	**Case vs. Control**			
			**OR**	**CI**	**X^2^**	***p*-Value**
G	66 (66%)	73 (73%)	0.72	0.39 to 1.31	1.15	*p* = 0.28
A	34 (34%)	27 (27%)	1.39	0.76 to 2.55		
(b)						
**Genotype**	**Case** ***N* = 50**	**Control** ***N* = 50**	**Case vs. Control**			
			**OR**	**CI**	**X^2^**	***p*-Value**
GG	22 (44%)	24 (48%)	0.85	0.39 to 1.87	0.16	*p* = 0.69
GA	18 (36%)	18 (36%)	1	0.44 to 2.26	0	*p* = 1
AA	10 (20%)	8 (16%)	1.31	0.47 to 3.66	0.27	*p* = 0.60
Total	50	50				
**Allele**	**Case** **(Freq)**	**Control (Freq)**	**Case vs. Control**			
			**OR**	**CI**	**X^2^**	***p*-Value**
G	62 (62%)	66 (66%)	0.84	0.47 to 1.5	0.35	*p* = 0.56
A	38 (38%)	34 (34%)	1.19	0.67 to 2.12		

## Data Availability

Data is contained within the article.

## Abbreviations

RSM	Recurrent spontaneous miscarriage
VEGF	Vascular endothelial growth factor
SNP	Single-nucleotide polymorphism
OR	Odds ratio
CI	Confidence interval
X^2^	Chi-square
